# Identification of HRH1 as an alternative receptor for SARS-CoV-2: insights from viral inhibition by repurposable antihistamines

**DOI:** 10.1128/mbio.01697-24

**Published:** 2024-07-22

**Authors:** Gang Zhong, Jinrong Li, Huiqing Wang

**Affiliations:** 1 Department of Orthopedic Surgery, West China Hospital, Sichuan University, Chengdu, China; 2 Trauma Center, West China Hospital, Sichuan University, Chengdu, China; 3 Department of Pediatrics, West China Second University Hospital, Sichuan University, Chengdu, China; 4 Key Laboratory of Birth Defects and Related Diseases of Women and Children (Sichuan University), Ministry of Education, Chengdu, China; Johns Hopkins University, Baltimore, Maryland, USA

**Keywords:** SARS-CoV-2, HRH1, viral entry, receptor

## Abstract

Numerous coreceptors have been shown to facilitate hACE2-dependent or hACE2-independent infection by SARS-CoV-2. A recent study published in *mBio* by Yu et al. showed that the histamine receptor H1 (HRH1) functions as an alternative receptor for SARS-CoV-2 via direct binding to viral spike proteins (F. Yu, X. Liu, H. Ou, X. Li, et al., mBio e01088-24, 2024, https://doi.org/10.1128/mbio.01088-24). Furthermore, they present compelling evidence that antihistamine drugs targeting HRH1 potently inhibit SARS-CoV-2 entry. This study highlights the therapeutic potential of repurposable antihistamines against COVID-19.

## COMMENTARY

Throughout the COVID-19 pandemic, SARS-CoV-2 underwent successive rounds of immune evasion and acquired enhanced transmissibility. Numerous mutations within the receptor-binding domain (RBD) of viral spike proteins have been identified. Nevertheless, different SARS-CoV-2 mutants can still efficiently infect target cells via the binding of the RBD to hACE2 receptors. Interestingly, various mutations in regions other than the RBD seem to influence the cellular tropisms of SARS-CoV-2. Specifically, mutations within the S1/S2 cleavage boundary of the Omicron spike attenuate furin- and TMPRSS2-mediated cleavage, thereby resulting in inefficient viral replication in the TMPRSS2-expressing lower respiratory tract (1, 2). In addition, numerous cellular proteins have been found to be coreceptors of SARS-CoV-2, including SR-B1, NRP1, DC-SIGN, L-SIGN, SIGLEC1, vimentin, ADAM9, CD147, AXL, KIM1, ASGR1, KREMEN1, LDLRAD3, CLEC4G, and TMEM106B (3–6). These cofactors function as both alternative receptors to enable hACE2-independent viral entry and accessory receptors to perform hACE2-dependent infection. The identification of these "universal" receptors may diversify the use of repurposed drugs targeting these receptors to prevent SARS-CoV-2 infection. However, it is crucial to exercise vigilant monitoring of potential adverse effects associated with direct targeting of these receptors.

A study published in *mBio* by Yu et al. revealed that the histamine receptor H1 (HRH1) is an alternative receptor of SARS-CoV-2 (7). They conducted an unbiased screening of a Food and Drug Administration (FDA)-approved drug library and discovered that all the antihistamine drugs against HRH1 also potently prevented entry of SARS-CoV-2 into susceptible cells. Furthermore, these drugs also prevented the entry of various viral mutants, demonstrating their broad-spectrum antiviral potential. The authors employed coimmunoprecipitation (Co-IP), immunofluorescence (IF), and surface plasmon resonance (SPR) assays to elucidate the underlying mechanism. Their results showed that HRH1 is directly bound to the N-terminal domain (NTD) of viral spike proteins, thereby enabling hACE2-independent viral entry. Interestingly, HRH1 also directly interacted with the hACE2 receptor and synergistically enhanced hACE2-dependent viral infection.

Antihistamine drugs have been commonly used to treat various allergies without severe side effects for decades. Therefore, manipulating HRH1 antagonists as inhibitors of SARS-CoV-2 entry could be safe. Yu et al. demonstrated that both acrivastine and triprolidine, two extensively employed antihistamines, effectively inhibited authentic SARS-CoV-2 infection with IC50 values of 2.694 µM and 0.938 µM, respectively. They further evaluated whether antihistamines could prevent authentic viral infection in animal models. Transgenic hACE2 mice were intravenously administered with acrivastine prior to the SARS-CoV-2 challenge. Their results showed that lung tissues obtained from SARS-CoV-2-infected mice in the control group exhibited profound pathological alterations characterized by collapsed alveoli, thickened alveolar septa, inflammatory cell infiltration, and dispersed N-expressing epithelial cells. Notably, acrivastine treatment effectively mitigated these pathological changes. These results suggest that HRH1 antagonists hold promise as prophylactic interventions for attenuating authentic SARS-CoV-2 infection in animal models, warranting further evaluation in clinical trials.

Based on the findings reported by Yu et al*.*, three models of HRH1/ACE2-mediated SARS-CoV-2 infection can be proposed. In cells exhibiting high expression levels of ACE2 and low expression levels of HRH1, SARS-CoV-2 enters target cells via direct binding of the viral spike protein to ACE2 receptors. Conversely, in cells expressing insufficient ACE2 but high amounts of HRH1, viruses can exploit HRH1 as an alternative receptor to infect these cells. However, in cells that express moderate levels of both ACE2 and HRH1, HRH1 binds to both ACE2 and the viral spike protein, which synergistically facilitates ACE2-mediated viral entry. Consequently, the manipulation of antihistamines in COVID-19 patients not only abolishes HRH1-mediated viral entry but also cripples ACE2-dependent viral infection. These scenarios are also applicable to other coreceptors, prompting us to screen efficacious and safe compounds for combating pathogenic diseases.

Although hACE2 has been demonstrated to be the predominant receptor of SARS-CoV-2, the protein expression levels of hACE2 within the respiratory tract are relatively lower than those within the intestine, kidney, and heart muscle. Consequently, multiple reports have shown that SARS-CoV-2 infection can also cause inflammatory responses within these tissues. Several reports have indicated that coreceptors facilitate both hACE2-independent and hACE2-dependent infections, which potentially explains why SARS-CoV-2 efficiently replicates in lung or trachea tissues. HRH1 is widely expressed within the respiratory tract, including nasal and lung tissues. The released histamines triggered by exogenous allergens bind to HRH1, resulting in the induction of allergic rhinitis and allergic lung responses. However, antihistamines can competitively bind to HRH1, which alleviates histamine-induced allergies. A study reported by Yu et al. showed that HRH1 also functions as an alternative receptor for SARS-CoV-2 (7). Both HRH1 antagonists and histamines prevent SARS-CoV-2 infection. Multiple large-scale screening studies have independently demonstrated that antihistamines, including clemastine, astemizole, azelastine, brompheniramine, and ebastine, can prevent SARS-CoV-2 infection (8, 9). Yu et al. evaluated the inhibitory effects of six first-generation antihistamines, namely, brompheniramine, clemastine, cyproheptadine, diphenhydramine, promethazine, and triprolidine, and five second-generation antihistamines, namely, acrivastine, astemizole, azelastine, desloratadine, and loratadine. Their results showed that all of these antihistamines potently inhibited SARS-CoV-2 infection. Their comprehensive study elucidated the anti-SARS-CoV-2 potential of different HRH1 antagonists, providing diverse options for SARS-CoV-2-infected individuals with distinct types of allergies.

Repurposed antihistamines have played diverse roles in combating COVID-19. Reports have shown that released histamines can elevate proinflammatory cytokines upon SARS-CoV-2 infection, thereby contributing to the development of a COVID-19-associated cytokine storm. Several clinical studies have revealed that cotreatment with antihistamines for COVID-19 patients significantly reduces the duration of hospitalization and prevents progression toward severe symptoms (10). Therefore, antihistamines are utilized as immunomodulators to alleviate symptoms of COVID-19. Interestingly, two other clinical studies have shown that antihistamines can also relieve long-term symptoms of COVID-19, suggesting their potential to treat long-term COVID-19 (11, 12). Yu et al. revealed that antihistamines prevent SARS-CoV-2 infection by directly uncoupling the binding of viral spike proteins to cellular HRH1 receptors. Collectively, these studies demonstrate that antihistamines can be utilized as both prophylactic interventions to inhibit SARS-CoV-2 infection and therapeutic countermeasures to alleviate COVID-19 symptoms, suggesting their viability as alternative pharmaceutical agents for combating COVID-19.
